# Dysfunctional Neurotransmitter Systems in Fibromyalgia, Their Role in Central Stress Circuitry and Pharmacological Actions on These Systems

**DOI:** 10.1155/2012/741746

**Published:** 2011-10-02

**Authors:** Susanne Becker, Petra Schweinhardt

**Affiliations:** Alan Edwards Centre for Research on Pain, Faculty of Dentistry, McGill University, Montreal, QC, Canada H3A 2B2

## Abstract

Fibromyalgia is considered a stress-related disorder, and hypo- as well as hyperactive stress systems (sympathetic nervous system and hypothalamic-pituitary-adrenal axis) have been found. Some observations raise doubts on the view that alterations in these stress systems are solely responsible for fibromyalgia symptoms. Cumulative evidence points at dysfunctional transmitter systems that may underlie the major symptoms of the condition. In addition, all transmitter systems found to be altered in fibromyalgia influence the body's stress systems. Since both transmitter and stress systems change during chronic stress, it is conceivable that both systems change in parallel, interact, and contribute to the phenotype of fibromyalgia. As we outline in this paper, subgroups of patients might exhibit varying degrees and types of transmitter dysfunction, explaining differences in symptomatoloy and contributing to the heterogeneity of fibromyalgia. The finding that not all fibromyalgia patients respond to the same medications, targeting dysfunctional transmitter systems, further supports this hypothesis.

## 1. Fibromyalgia as a Stress-Related Disorder

Fibromyalgia is characterized by heightened pain perception, including widespread hyperalgesia, in particular to deep-pressure stimuli, enhanced temporal summation, and reduced pain-inhibiting effects of heterotopic noxious stimulation (often termed diffuse noxious inhibitory control, DNIC) [1]. Fibromyalgia has often been described as a stress-related disorder, and altered stress systems have been viewed as causal for pain and other symptoms experienced in this condition [2]. The body's two stress systems, the hypothalamic-pituitary-adrenal (HPA) axis and the sympathetic nervous system, are indeed altered in fibromyalgia [1]; however, results on the specific changes are heterogeneous. For both systems, hyper- as well as hypoactivity in basal functioning and acute stress responses has been reported (e.g., [3–8]). Concerning the HPA axis, it has been suggested that prolonged periods of stress associated with heightened basal tone and exaggerated acute stress responses (hyperreactivity) are followed by the development of a hyporeactive HPA axis, thus potentially explaining inconsistent findings regarding the HPA axis [9].

Stress increases the risk of developing fibromyalgia, dependent on different predispositions (e.g., genetic makeup and gender) [2]. However, it is still unclear which physiological processes mediate the relationship between experienced stress and the development of fibromyalgia. Changes in the autonomic and HPA stress systems are often considered as such mediators, with chronic stress exposure altering the functioning of these stress systems, causing fibromyalgia symptoms [2, 10]. In line with this view, the cardinal symptom of the condition seems to be related to alterations of the HPA axis: reported levels of clinical pain have been shown to be associated with concentrations of corticotropin-releasing hormone (CRH) in the cerebrospinal fluid (CSF) [11] and to salivary cortisol levels [12].

Nevertheless, prospective studies are scarce and available results do not allow conclusions on causal relationships [13]. In addition, in contrast to pain, other prominent symptoms associated with fibromyalgia, such as fatigue, depressivity, and perceived stress, appear not to be related to measures of HPA axis function [11, 12]. It is, therefore, conceivable that fibromyalgia symptoms are associated with altered autonomic and HPA axis stress systems but that these altered stress systems do not necessarily cause the symptoms. Stress-related changes in other physiological systems, for example, neurotransmitter systems, might be additionally involved in symptom development. Further, stress-related changes in such other systems may develop in parallel to changes in the autonomic and HPA axis systems or even precede them, thereby contributing to or causing fibromyalgia symptoms.

In support of these considerations, some evidence suggests that dysfunction of the body's autonomic and HPA axis stress systems are related to some of the risk factors for developing fibromyalgia, such as early-life stress [14] rather than playing a causal role in the pathogenesis of fibromyalgia. For example, salivary cortisol levels in a cross-sectional study were shown to differ depending on the presence or absence of early-life trauma (physical or sexual abuse) but did not differentiate between fibromyalgia patients and healthy controls [12]. Similarly, CRH concentrations in the CSF have been shown to be strongly related to the presence or absence of early-life trauma (physical or sexual abuse) [11]. Regarding the sympathetic system, evidence in healthy volunteers suggests that reduced heart rate variability may be a predisposing factor for the development of fatigue, pain, and depressive symptoms rather than the underlying cause of these symptoms [15].

## 2. Dysfunctional Transmitter Systems in Fibromyalgia

**Figure 1:**
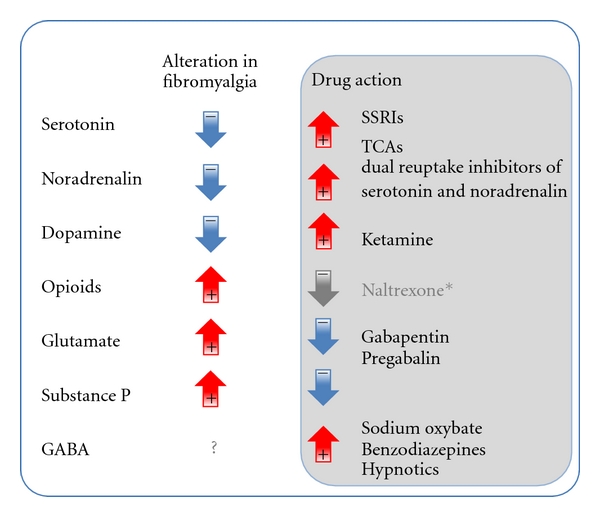
Alterations in transmitter systems found in fibromyalgia patients in terms of increased or decreased activity and action of drugs used in fibromyalgia on these transmitters systems in terms of activity increase or decrease. *The positive effect of naltrexone, an opioid antagonist, on fibromyalgia symptoms is suggested to be mediated through disinhibiting effects on HPA axis activity, rather than by its effect on the opioid system (cf. [16–18]).

Cumulative evidence points at alterations in neurotransmitter systems in fibromyalgia (see Figure 1), which is interesting because the main symptoms of fibromyalgia, that is, heightened pain perception, fatigue, sleep disturbances, and depressive as well as anxiety-related symptoms, are closely linked to these neurotransmitters.

The key symptom and main diagnostic criterion for fibromyalgia is chronic widespread pain. Several neurotransmitters and modulators are substantially involved in pain processing. For example, central serotonin and noradrenalin are important in endogenous pain inhibitory pathways [19, 20] and serotonin plays also an essential role in descending pain facilitation via the 5HT_3_ receptor [21, 22]. Substance P is a neuropeptide that is important for spinal nociception. It coexists with the excitatory neurotransmitter glutamate in primary nociceptive afferents [23] and causes sensitization of dorsal horn neurons [24, 25]. Not surprisingly, glutamate itself plays an important role in nociception, as it has excitatory and sensitizing effects [26]. In addition, glutamate has some inhibitory effects in descending pain pathways [21]. Although it has to be acknowledged that the exact effects and modulatory actions of these transmitters depend on receptor subtypes and CNS site [21, 22], serotonin, noradrenalin, substance P, and glutamate have been shown to be altered in fibromyalgia in ways that could explain patients' increased pain sensitivity. CNS levels of serotonin and noradrenalin appear to be lowered, indicated by decreased levels of metabolites in the CSF and of serotonin and noradrenalin in blood [27–30], possibly contributing to dysfunctional descending pathways and resulting in attenuated descending inhibition (cf. [31]). CSF concentrations of substance P and glutamate have been repeatedly found to be increased in fibromyalgia patients [32–34]. With respect to glutamate, proton magnetic resonance spectroscopy studies could show that this neurotransmitter is elevated in pain processing regions such as the insula, amygdala, and cingulate cortex [35–39]. Supporting the hypothesis that a hyperactive glutamate system contributes to increased pain sensitivity, and maybe other symptoms of fibromyalgia, elevated glutamate levels in the insular cortex have been observed to be correlated with low pressure pain thresholds [39] as well as with high scores on the fibromyalgia impact questionnaire (FIQ, [40]) [37]. 

Similar to serotonin and noradrenalin, dopamine activity has been demonstrated to be attenuated in fibromyalgia (see [41] for review): CSF levels of dopamine [28] and presynaptic dopamine function are reduced (examined with positron emission tomography (PET)) [42], and dopamine responses to acute pain are diminished in fibromyalgia patients [43]. Since inactivation of D2 receptors has been shown to lead to hyperalgesia [44], these findings may suggest that dysfunctional dopaminergic neurotransmission contribute to patients' pain symptomatology.

Particularly important for the endogenous control of nociception are endogenous opioids, as they decrease transmission of nociceptive signals in several pathways and nuclei [21, 45]. Counterintuitively, opioid activity appears to be increased in fibromyalgia as indicated by increased CSF and blood serum opioid levels [46], upregulation of opioid receptors [47], and reduced cerebral mu-receptor binding at rest (indicative of increased release) [48]. It is not readily conceivable how an overactive opioid system would contribute to fibromyalgia symptoms. Indeed, elevated levels of opioids might be a consequence of pain, rather than a cause, since similar findings have been obtained in other chronic pain conditions [49, 50]. Nevertheless, mu-opioid receptor binding potentials have been found to be negatively correlated with measures of affective pain in fibromyalgia [48], perhaps explaining the emotional connotation of pain in fibromyalgia. Another important neurotransmitter of antinociception is GABA [51], the main inhibitory neurotransmitter in the CNS. Although direct investigations are not yet available, pharmacological studies have shown a certain effectiveness of GABAergic agents for pain, sleep, and fatigue, suggesting that this inhibitory neurotransmitter system might also be impaired in fibromyalgia.

In addition to increasing pain sensitivity, alterations in serotonin, noradrenalin, and substance P may contribute to disturbances in sleep or mood in fibromyalgia patients. Serotonin and noradrenalin are strongly associated with circadian rhythms (see [52] for review), and serotonin is recognized as a mediator of deep sleep [53]. Moreover, a deficient serotonin system is strongly associated with major depression [54]. Increased levels of intracerebral substance P have been associated with increased anxiety-like behavior in animals [55], and accordingly, NK1-receptor blockade (NK-receptors are the receptors for substance P) is associated with reduced anxiety [56].

## 3. The Role of Altered Transmitters in Stress Systems

Dysregulated neurotransmitter systems have been suggested to play a role in the etiology and pathogenesis of stress-related pathologies including fibromyalgia (cf. [57, 58]). For example, deficient noradrenergic modulatory function is hypothesized to increase the vulnerability to stress-related pathology [58]. In line with this hypothesis, all of the neurotransmitters systems found to be altered in fibromyalgia exert influences on the sympathetic nervous system or the HPA axis stress system (see [52, 59] for review; see Table 1).

Serotonin and noradrenalin have been shown to have a mainly excitatory influence on acute stress responses and both are key in circadian rhythm of the HPA axis [52, 59–61]. Dopamine has excitatory influences on the basal tone of the HPA axis and enhances acute stress responses, as demonstrated in various animal and human studies (e.g., [62–64]). Another excitatory neurotransmitter in CNS stress circuits is glutamate even though glutamate is present also in inhibitory stress circuits [52, 65–67]. It is hypothesized that an optimal “glutamate tone” is required, whereby too little or too much results in HPA activation [52]. GABA and substance P both inhibit HPA axis functioning: they have a tonic inhibitory influence on the HPA axis and terminate acute HPA stress responses (GABA [68–70]; substance P [59, 71, 72]). Evidence suggests that opioids diminish stress-induced autonomic stress responses [57, 73], but for the HPA axis, both inhibitory and excitatory effects have been found [74, 75], presumably depending on receptor subtypes and type of stressor [74–76].

The transmitter disturbances observed in fibromyalgia could readily explain hyporeactivity of both stress systems, as found in fibromyalgia (see above; [1]). Transmitters that regulate circadian rhythm and enhance acute stress responses such as serotonin, noradrenalin, and dopamine are reduced in fibromyalgia, while substance P, which inhibits basal tone and acute responses of the HPA axis, is increased. Similarly, opioids, which are increased in fibromyalgia, inhibit acute sympathetic and HPA axis stress responses.

In contrast, these transmitter aberrations cannot easily explain a hyperactivity of the stress systems, which has equally been shown in fibromyalgia [1]. This might be because the view presented in the preceding paragraph is very simplistic. The specific effect of a neurotransmitter may be only weakly related to its global level (which is the measure often obtained in human studies) but depends on factors such as receptors subtype, brain region, concentration relative to other neurochemicals, and the type of stressor. For example, evidence suggests functional differences of serotonin receptor subtypes in HPA axis regulation [61, 77], and the modulatory function of serotonin appears to be dependent on specific brain regions and stressors [60]. The same has been suggested for dopamine [63, 78–80] and glutamate [66, 81–84]. Similarly, the differential inhibitory and excitatory effects of opioids have been suggested to be due to different opioids acting through different opioid receptors in addition to a dependence on stimulus conditions [74–76].

The situation gets even more complicated if one takes into account changes in neurotransmitter functioning due to chronic stress that in turn affect sympathetic and HPA axis stress responses. Chronic stress leads to attenuated HPA axis responses that are mediated by serotonergic neurotransmission, in contrast to the serotonin-mediated increase of acute HPA axis responses under normal conditions [52, 60, 61]. Noradrenalin release seems to be increased by chronic stress through sensitized noradrenergic neurons, leading to enhanced autonomic and HPA axis excitability [52, 58, 85–87]. In otherwise healthy organisms, the experience of chronic stress has been demonstrated to result in increased as well as decreased dopaminergic activity depending on receptor subtype and brain region [88]. In general, dopaminergic responses to stressors seem to be enhanced after exposure to chronic stress [78], which could lead to hyperreactive stress systems, since these systems are excited by dopamine. In accordance with increased levels of endogenous opioids and substance P found in fibromyalgia, opioids [57, 89] and substance P [52, 90] have been found to be increased in response to chronic stress, leading to an attenuation of HPA axis reactivity. Results on changes of glutamate and GABA systems due to chronic stress are not conclusive: some glutamate [91–93] and GABA [94–96] receptors subunits are upregulated, while others are downregulated with chronic stress depending on brain regions.

These diverse results strongly suggest that chronic stress does not affect transmitters and stress systems uniformly. In fact, the diversity of the results on chronic stress-induced changes in transmitter functioning favoring in some instances hypoactive, and in other instances, hyperactive stress systems are reminiscent of the range that is found regarding the activity of the stress systems in fibromyalgia patients. So perhaps whether the sympathetic nervous system and/or the HPA axis is hyper- or hypoactive in a given individual depends on the ratio of dysfunctions in different transmitter systems, rather than absolute transmitter levels. For example, hyperactivity of the HPA axis could be associated with alterations in glutamate and opioids that are more pronounced than changes in serotonin, noradrenalin, and dopamine. The different transmitter dysfunctions may also change as a function of time, which could then contribute to stress systems alterations that are not constant over time (cf. [9]). Individual patients might exhibit varying degrees and types of transmitter dysfunction, and indeed, fibromyalgia patients are recognized to be a heterogeneous group. Accordingly, categorization of fibromyalgia patients into subgroups has been suggested. Generally, fibromyalgia patients are subdivided into a group with a predominant pain phenotype (strong hyperalgesia) without or only mild related psychopathological findings and into patients with (major) depression although different ways of categorizing and different numbers of subgroups have been suggested [97–99]. In any case, most studies on dysfunctional neurotransmitters as well as on stress systems in fibromyalgia have not taken any subcategorization into account even though it seems reasonable to assume that these subgroups differ not only with respect to their symptoms but also regarding the mechanisms underlying the condition. Considering that transmitter alterations seem to be strongly related to symptoms, it seems conceivable that subgroups of fibromyalgia patients are characterized by different transmitter alterations and that the ratio of dysfunctions in different transmitter systems varies between subgroups. The observation that not all fibromyalgia patients respond to the same medications (Figure 1). Further supports the notion that subgrouping might be important in studies on fibromyalgia.

## 4. Pharmacological Interventions in Fibromyalgia Targeting Dysfunctional Transmitter Systems

Pharmacological compounds that raise serotonin and noradrenalin concentrations such as tricyclic antidepressants (TCAs) and dual reuptake inhibitors of serotonin and noradrenalin are relatively effective treatments of fibromyalgia, improving mainly pain, sleep, and fatigue although not in all patients (see [100–102] for review). Interestingly, the beneficial effects of these medications are independent of effects on mood (e.g., [103, 104]). Selective serotonin reuptake inhibitors (SSRIs) are less effective compared to TCAs and dual reuptake inhibitors. Moreover, newer SSRIs (e.g., citalopram), which are even more selective for serotonin reuptake inhibition, appear to be even less effective compared to older SSRIs (e.g., fluoxetine and paroxetine) [101]. Taken together, reuptake inhibition of noradrenalin seems to be more important compared to reuptake inhibition of serotonin. Therefore, it would be interesting to investigate the effects of selective noradrenalin reuptake inhibitors (e.g., reboxetine) on fibromyalgia symptoms, which has not yet been done to the best of our knowledge. Moreover, antidepressants such as TCAs or SSRIs, dampen HPA axis activity in patients with major depression [105]. If this was also true in patients with fibromyalgia, another factor for the choice of medication would be the HPA axis activity status of an individual patient.

A very interesting finding is that 5-HT_3_ receptor *anta*gonists are effective in fibromyalgia patients with a primary pain phenotype (without depression) but not in fibromyalgia patients with depression [97]. 5-HT_3_ antagonists act antihyperalgesic probably through a reduction of descending pain facilitation [22]. The finding that some fibromyalgia patients respond to 5-HT_3_ antagonists does not necessarily fit with the finding of decreased serotonin activity in fibromyalgia. So, perhaps fibromyalgia patients with a primary pain phenotype, who respond to 5-HT_3_ antagonists, do not have decreased serotonin levels and only those in whom depressive symptoms dominate the clinical picture would show decreased serotonin levels when subgrouped. However, one has to be cautious with this hypothesis, because serotonin concentrations could potentially vary across different CNS sites. 

Glutamate and substance P disturbances in fibromyalgia might be targeted by pregabalin. Pregabalin binds to the *α*_2_ *δ* subunit of voltage-dependent calcium channels and decreases the release of a variety of neurotransmitters, including glutamate and substance P [106, 107] by reducing the calcium influx into nerve terminals. Pregabalin is effective particularly for pain and sleep. Interestingly, only small or no effects on anxiety symptoms have been found in fibromyalgia [108–112] although pregabalin is known to have anxiolytic effects [113] and is approved by the European Union for the treatment of anxiety disorders. 

Other pharmacological treatments have been tested in fibromyalgia patients but evidence is weaker [100]. Ketamine is an interesting molecule: typically conceived as a NMDA receptor antagonist, it has recently been demonstrated to act mainly as a D2 dopamine receptor agonist in low doses [114]. Such low doses lead to reductions in experimental and clinical pain in approximately half of the tested fibromyalgia patients [115–117]. A study on the NMDA receptor antagonist dextromethorphan failed to demonstrate positive effects on experimental pain in fibromyalgia patients [118], suggesting that the beneficial effects of ketamine might indeed be related to its dopaminergic properties. The effects of ketamine on the HPA axis vary: while high doses consistently increase HPA axis activity, doses comparable to those used in fibromyalgia have been found to enhance or dampen effects HPA axis functioning (e.g., [119–121]).

Interestingly, naltrexone, an opioid antagonist, has shown some beneficial effect on fatigue and perceived stress in fibromyalgia patients [122]. Since naltrexone disinhibits HPA activity [16–18], it could be postulated that it might be particular effective in patients with low HPA axis activity and in whom fatigue is the predominant symptom rather than pain.

Disturbances of GABAergic neurotransmission have not yet been directly investigated in fibromyalgia. Nevertheless, a certain effectiveness of sodium oxybate (*γ*-hydroxybutyrate) (e.g., [123–125]), which acts as a GABA_B_ receptor agonist, benzodiazepines [126], which enhance the effect of GABA, and (nonbenzodiazepine) hypnotics [127–129], which act as GABA_A_ receptor agonists, for pain, sleep, and fatigue has been observed. Benzodiazepines and hypnotics that act at GABA_A_ receptors dampen HPA axis activity (e.g., [130–133]). Interestingly, zolpidem, which acts selectively at the GABA_A_ *α*_1_ receptor subunit, has been shown to enhance HPA axis activity [131, 132]. Presumably, this differential effect depends on the drug's effect on a specific GABA receptor subunit and the net effect of the nonselective drugs results from by action on different receptor subunits [131, 132]. It would be interesting to investigate whether the effects of selective and nonselective drugs targeting GABAergic neurotransmission are different in fibromyalgia patient subgroups with hypo- or hyperactive stress systems.

## 5. Stress-Induced Changes in Transmitter Systems as a Pathogenic Factor in Fibromyalgia?

Because transmitter changes seem to be closely related to fibromyalgia symptoms and could, at least partly, explain alterations observed in the HPA axis as well as the sympathetic system, dysregulated neurotransmitter systems may play a pathogenic role in fibromyalgia (cf. [57, 58]). Indeed, chronic stress induces changes in relevant neurotransmitters, as discussed above. In this theoretical framework, stress-induced changes in transmitter systems would cause pain as well as other symptoms in fibromyalgia and contribute to the observed changes in the sympathetic as well as HPA stress system. In addition, chronic stress also directly modifies the HPA axis and the autonomic nervous system, and the stress systems are likely to influence the transmitter systems. Consider, for example, substance P: chronic stress leads to an increase in substance P [52, 90] and can cause a hyporeactivity of the HPA axis. But because substance P itself inhibits the HPA axis, the causal relationship remains unclear. Different scenarios are conceivable: the first one is, the “serial stress system-based view” in which changes in the functioning of the autonomic and HPA axis stress systems, as a result of chronic stress, cause fibromyalgia symptoms and alter transmitter systems. In this scenario, changes in the stress systems precede and cause fibromyalgia symptoms and dysfunctional transmitter systems, considering dysfunctional transmitters systems not as causally relevant for the pathogenesis of fibromyalgia. The second scenario is, the “serial transmitter-based view” in which changes in transmitter functioning, as a result of chronic stress, cause fibromyalgia symptoms and alter autonomic and HPA axis stress systems. In this second scenario, dysfunctional stress systems are not considered as causally relevant for the pathogenesis of fibromyalgia in that dysfunctional transmitter systems precede and cause fibromyalgia symptoms and altered stress systems. Lastly, we have the “parallel view” in which chronic stress is considered to cause dysfunctional transmitter as well as autonomic and HPA axis stress systems in parallel. Neither changes in transmitter systems nor in stress systems precede each other, but changes in the systems interact and both dysfunctional transmitter and stress systems finally cause fibromyalgia symptoms. 

The current evidence does not conclusively favor one model. Longitudinal studies in fibromyalgia that track the development of disturbances in transmitters as well as stress systems over time would be important in order to test these models. Further, any study on the topic is likely to substantially benefit from subcategorizing fibromyalgia patients. Similarly, treatment studies should investigate well-defined subgroups of patients, ideally selected based on specific biochemical alterations that are hypothesized to be impacted by the specific therapy. Although—or maybe because—this is a long “to-do” list, it has to be acknowledged that research in recent years has already made great advances in uncovering CNS alterations and potential mechanisms that might contribute to the complex clinical phenomenon of fibromyalgia.

## Figures and Tables

**Table 1:** Overview of the effects of the neurotransmitter systems found to be altered in fibromyalgia on HPA axis activity and the effects of drugs used in fibromyalgia, targeting these transmitter systems, on HPA axis activity. Information on effects on the sympathetic nervous system is not included, because results are scarce. The specific effect of the transmitters depends on different aspects of HPA axis activity, that is, basal tone and circadian rhythm or acute stress responses. In addition, under conditions of chronic stress, the transmitter effects on the HPA axis are often altered. The table represents a simplistic summary of the evidence found on transmitter actions on HPA axis activity. Despite a vast number of studies, the precise mechanisms of neurotransmitters on HPA axis functioning remain only incompletely understood [52]; transmitter actions depend on receptor subtypes, brain regions, and type of stressor.

Transmitter system	Effect on HPA axis activity in terms of		HPA axis activity under chronic stress*	
	basal tone and circadian rhythm	acute stress responses		Effect of drugs on HPA axis activity*
Serotonin	excitatory (↑) and inhibitory (↓)	excitatory (↑)	inhibitory (↓)	excitatory (↑) and inhibitory (↓)
Noradrenalin	excitatory (↑) and inhibitory (↓)	excitatory (↑)	excitatory (↑)	excitatory (↑) and inhibitory (↓)
Dopamine	excitatory (↑)	excitatory (↑)	excitatory (↑)	?
Opioids	excitatory (↑)	inhibitory (↓)	inhibitory (↓)	excitatory (↑)
Glutamate	?	excitatory (↑)	?	excitatory (↑) and inhibitory (↓)
GABA	inhibitory (↓)	inhibitory (↓)	excitatory (↑)	?
Substance P	inhibitory (↓)	inhibitory (↓)	inhibitory (↓)	?

*Chronic stress as well as drugs have differential effects on basal tone, circadian rhythm, and acute responses to stress but most studies do not differentiate these aspects. Accordingly, this table does not differentiate in these instances.
